# The Interplay between Emotional processing and Error‐monitoring as a relevant mechanism in the Self‐awareness loop ‐ Evidence from the ADNI cohort

**DOI:** 10.1002/alz70857_103639

**Published:** 2025-12-25

**Authors:** Katia Andrade, Patrick Maloyan, Dimitrios Pantazis, Swapna Mahurkar‐Joshi, Takfarinas Medani, Anand A Joshi, Richard Leahy

**Affiliations:** ^1^ Institute of Memory and Alzheimer's Disease (IM2A), Salpêtrière University Hospital, Paris, Paris, France; ^2^ MIT, Boston, MA, USA; ^3^ Massachusetts Institute of Technology, Cambridge, MA, USA; ^4^ University of California Los Angeles, Los Angeles, CA, USA; ^5^ University of Southern California, Los Angeles, CA, USA

## Abstract

**Background:**

Lack of awareness of deficits, known as anosognosia, is a very impressive neuropsychiatric symptom of Alzheimer's disease (AD). It often appears in the early stages of the disease and worsens over time.

**Method:**

According to our dual‐path hypothesis, the occurrence of anosognosia in AD would depend on a critical failure of the error‐monitoring system, reflecting either (i) local alterations in the cingulate cortex (direct pathway), with particular emphasis on its anterior and posterior parts (ACC and PCC, key brain regions at the origin of error‐related potentials, ErrP); and/or (ii) an inability to link error‐monitoring and emotional processing for adaptive behavior due to impairments in the latter system (indirect pathway), mainly affecting the orbitofrontal cortex (OFC) and the amygdala. This study focused on a subgroup of the longitudinal ADNI cohort characterized by 125 subjects who progressed from mild cognitive impairment, MCI, to AD. Our main goal was to investigate which brain structures and specific areas, without any a priori out of a total of 360, contributed the most to unawareness of deficits over time.

**Result:**

**The results show that self‐awareness deterioration correlated with cortical thickness changes in specific areas of two brain structures: the OFC (area p32, right hemisphere, *p* <0.01, corrected; a25, left hemisphere, *p* = 0.051, corrected) and the ACC (area a24, left hemisphere, *p* = 0.051, corrected)**.

**Conclusion:**

These findings strongly support our dual‐path model for the emergence of anosognosia in AD, bringing to the front the relevance of the indirect pathway, linking emotional processing to error‐monitoring, in the self‐awareness loop.

